# Ophthalmology

**Published:** 1895-03

**Authors:** F. R. Cross


### OPHTHALMOLOGY.

A large proportion of individuals are, even in the present day, and despite increasing knowledge of the modes of prevention and cure of disease of the eye, losing their sight from causes which are absolutely preventible. Of the inmates of Blind Asylums from a quarter to a half (according to various statistics) have been the subjects of infectious eye diseases: blenorrhœa of the new-born and of adults, trachoma, granular ophthalmia, and allied disorders due to overcrowding, foul air, and want of cleanliness. Very much ought yet to be done in all these by prophylaxis, particularly by the better education of midwives as to the danger of the slightest cold or inflammation in the eyes of a newly-born child.

It has been stated that in America blindness is on the increase, as compared with the population, one of the principal causes being ophthalmia neonatorum. New York and several other States[1] have passed laws with the object of prophylactic treatment of this disease, by imposing a severe penalty upon the midwife or nurse who does not report to the recognised authority any case of infantile ophthalmia directly definite signs of inflammation have shown themselves.

Just lately, after a paper read by Dr. Morrison Ray,[2] in the Kentucky State Medical Society, the following resolution was passed: "Whereas there are in this State nearly one thousand persons blind from ophthalmia neonatorum, and this . . . is largely preventible, being due to the neglect of nurses and midwives . . . there shall be presented to the next

[1] Lucien Howe, *Am. J. Ophth.*, vol. xi., 1894, p. 20.
[2] *Am. Pract. & News*, vol. xviii., 1894, p. 302.

General Assembly for their enactment a suitable law, with proper penalties, requiring nurses and midwives to report . . . all cases of inflamed, swollen, or reddened eyes occurring in infants under their charge within six hours after the discovery of such conditions."

In 1884 the Ophthalmological Society of the United Kingdom[1] held important communications with the Local Government Board, with the intention of introducing some practical means by which ophthalmia in infants should be prevented, or when occurring should be at once submitted to effective treatment. In Ireland the authorities took action without delay, in accordance with the advice given by the Society; but in England the usual official difficulties were arrayed against the proposal, and no further progress has since been made, though the French, Austrian, and Hungarian Governments had issued edicts on this subject previous to the date mentioned.

In all forms of conjunctivitis, cleanliness by means of frequent thorough washings of the everted mucous membrane is the first essential for cure. In the simple cases, which are by far the commonest of all diseases of the eyes, nothing else is required than a liberal use of boric acid solution (which is improved by the addition of common salt) as a spray or a douche in an eye-cup at a temperature of about 60° F. A three per cent. solution of peroxide of hydrogen has been much extolled by Dr. Lautenbach[2] as a wash for the conjunctiva. He mentions three trustworthy brands of manufacture, and considers that the free acid usually left for preservation of the fluid is objectionable. Tepid water and Castile soap are valuable for cleaning the lids. Wade[3] prefers a lotion of carbolic acid (which is anæsthetic) with boric acid and glycerine.

In chronic cases of conjunctivitis, massage with castor oil, and in the follicular variety acetate of lead ointment, are recommended; and for trachoma, trituration with alum, sulphate of copper, or modified nitrate of silver stick. Dr. Kenneth Scott, who has had exceptional opportunities in Egypt of treating trachoma, formerly used a four per cent. solution of perchloride of mercury, with an eyedrop of a quarter per cent. At the Bristol meeting of the British Medical Association, he advocated, as an improvement, the use of cyanide of mercury, which is much less irritating and quite as efficacious. A four per cent. solution of the cyanide should be daily brushed on the everted lids, and a quarter per cent. solution used as an eye-drop three times a day. A slower cure is obtained by painting a one per cent. solution, and using as a drop one in 1,000. Warm weather seems distinctly favourable to a cure. Mr. Stephenson advocates a one per cent. solution of the perchloride, sulphate of copper crystal, and "expression." He considers trachoma to

[1] *Tr. Ophth. Soc. U. Kingdom*, 1885. [2] *Am. J. Ophth.*, vol. xi., 1894, p. 44.
[3] *Therap. Gaz.*, vol. xviii., 1894, p. 720.

be a specific form of follicular conjunctivitis, due to the irritation of a specific organism. Many authorities still consider sulphate of copper crystal the best application.[1]

In blenorrhœa, all authors agree as to the value of nitrate of silver; but there is much difference in the strength of its application. The two per cent. solution recommended by Credé for disinfection of the eyes of newborn children is the strength generally applied for all kinds and stages of purulent ophthalmia. The value of this remedy is probably largely due to its antiseptic properties. Weeks found that a two per cent. solution destroyed the vitality of staphylococcus pyogenes aureus and typhoid bacillus in eight seconds, and that a one per cent. solution killed them in twelve seconds. Solutions of one or two grains to ℥j are by some considered effectual as eyedrops, while several American oculists use sixty grains to ℥j, and the solid caustic is a constant remedy. Taken in time ophthalmia neonatorum is readily cured; but progress of a few days makes the disease much more serious. In the gonorrhœal ophthalmia of adults, prognosis must be very guarded. In one of the largest and best conducted eye clinics, out of 250 eyes affected with this disease 30 were lost, and the cornea was badly damaged in 74.

While nitrate of silver is of such value in purulent conjunctivitis, its application in the treatment of membranous and diphtheritic conjunctivitis is contra-indicated. Valude[2] also condemns cold compresses and sublimate lotions. He relies on warm sprays of weak non-irritating antiseptics, and he prefers a solution of extract of opium in sterilised water and glycerine. He also applies iodoform ointment to the *cul-de-sac*, treating corneal complications as they occur, and rigidly maintaining the general health. Three cases are reported of successful treatment of diphtheritic conjunctivitis by antitoxin injections. The nature of the cases was proved by bacteriological examinations. Dr. Coppez[3] cured a child one year old by one injection of Behring's serum. In twenty-four hours she was "literally transformed:" the eyelids had recovered their suppleness, the infiltration was disappearing, the false membrane had come away, and in four days the cure was complete. Jessop[4] reports two cases: (1) A boy nineteen months old received three injections of Klein's antitoxin (ʒss. each); the membrane disappeared in five days. (2) A boy eight months old had severe implication of the ocular and nasal mucous membranes, with enlargement of the parotid lymphatic glands. Two injections of Klein's antitoxin (ʒss. each) were given, and the membrane disappeared in five days. It was uncertain how long the membrane had been present before treatment was commenced. There are probably no cases on record of diphtheritic con-

[1] *Brit. M. J.*, 1894, vol. ii., p. 589, contains a paper by Juler, and a discussion on the general subject of ophthalmia. [2] *Ann. d'ocul.*, Fév., 1894.
[3] *Brit. M. J.*, 1894, vol. ii., p. 1340. [4] *Lancet*, 1895, vol. i., p. 347.

junctivitis so rapidly and completely cured, and in them no local treatment, excepting distilled water, had been used.

The importance of frequent sterilisation of the eyedrops in general use, such as atropine, cocaine, eserine, &c., which become infested with micro-organisms, has been emphatically shown by Dr. G. E. de Schweinitz and his brother.[1] They examined the pipettes and collyria in use in the clinic and found micrococci, bacilli, and fungi present. Among them were the micrococcus prodigiosus, proteus vulgaris, and bacillus implexus, which not only cause toxic conjunctivitis and serpentine or sloughing ulcers on an abraded cornea, but when reaching the aqueous chamber set up purulent inflammation of iris and deeper coats of the eyeball.

In the Bristol Eye Hospital we have for some time been in the habit of sterilising all dressings and applications for operation cases as well as the alkaloidal collyria in the out-patients' department.

F. R. CROSS.

### Reviews of Books.

[1] *Therap. Gaz.*, vol. xviii., 1894, p. 443.

